# Catalyzing collaboration through intentional design: the Teaming for Biomedical Digital Twin (Teaming4BDT) conference

**DOI:** 10.3389/fpsyg.2026.1781471

**Published:** 2026-03-18

**Authors:** L. Michelle Bennett, Zhen Xiao

**Affiliations:** 1 L. M. Bennett Consulting LLC, Potomac, MD, United States; 2 Truvante LLC, Lorton, VA, United States

**Keywords:** biomedical, collaboration, digital twin, interactive conference design, interdisciplinary, team development, teaming

## Abstract

Scientific conferences traditionally focus on knowledge dissemination through presentations, panel discussions, and poster sessions, with limited emphasis on active collaboration formation. We describe an ambitious conference design model that intentionally integrated team science principles to spark collaborative conference outcomes. The 2024 IMAG/MSM Teaming for Biomedical Digital Twin (Teaming4BDT) Meeting, was a three-day hybrid conference with over 200 participants that integrated didactics, presentations, and working group sessions focused on the opportunities and challenges of biomedical digital twins (NASEM definition and model) with structured team formation activities. Through dual perspectives—conference design and participant experience—we examine how deliberate design elements facilitated the formation of over 10 interdisciplinary teams and detail the journey of one team from formation through sustained collaboration.

## 1 Introduction

The 2024 Interagency Modeling and Analysis Group-Multiscale Modeling (IMAG/MSM) Consortium hosted a meeting centered on the NASEM definition of a “digital twin” and its application to biomedical research. The primary objective was to “promote, empower, and facilitate the understanding, collaboration, creation of tools, and infrastructure for biomedical digital twins (BDT)” (NASEM, 2024; Multiscale Modleing Consortium, 2024). Organizers were interested in promoting the essential role interdisciplinary collaboration will play in the design, development, and implementation of BDTs. To this end, in addition to spontaneous collaborative opportunities presenting themselves during breaks and lunch, intentional team formation was integrated into the meeting design.

Built upon the NASEM foundation, “Teaming for Biomedical Digital Twins” (Teaming4BDT) aimed to catalyze a community of practice across academia, industry, medicine, and government, translating theoretical models into systems that can predict or mitigate disease. Several key objectives:

- *Education*: establishing a fundamental understanding of the NASEM definition.
- *Application*: identifying and overcoming the biological and behavioral hurdles.
- *Collaboration*: convening experts across disciplines to share best practices and co-design BDT concepts of high interest.
- *Refinement*: providing collaborative space for participants to pitch ideas and receive feedback to improve their conceptual models.

These objectives align well with the integration of collaborative principles into the meeting design. Not only was there an opportunity to educate on the importance of team science principles and how they were applicable to BDT design. It was possible to have participants apply that knowledge in a team setting, addressing a real-world problem of their choosing, and responding to feedback from their peers.

To fully understand the potential impact and mechanics of Teaming4BDT, the following examines the conference through two distinct lenses: design (LMB) and participant (ZX). By considering the “before, during, and after” of the event from two perspectives, we gain a comprehensive view of how interdisciplinary collaboration integrated within the context of a conference can move from a concept to functional reality.

## 2 From vision to practice: parallel journeys

### 2.1 Before the meeting

#### 2.1.1 Designer view: conference design

Designing the Teaming4BDT Conference began by considering the goal that participants would depart with practical knowledge necessary to design a BDT, have networked with experts across disciplines, and have tools and templates to support them in that work. To succeed, the workshop design needed to draw from well-established principles from the learning sciences.

People remember and apply more when they move beyond passive listening and engage in discussion, reflection, and hands-on application. For that reason, we structured the days with short didactics, peer presentation, group discussions, collaborative problem-solving, and opportunities to apply concepts to real challenges. This progression—from exposure to interaction to practice—supports deeper understanding, stronger retention, and more rapid movement from theory to practical skill. Decades of research on active learning demonstrate

- Understanding and retention increase when learners generate explanations, solve problems, and interact meaningfully with content (Freeman et al., 2014).
- Applying a concept, receiving feedback, and reflecting on the experience reinforce mastery far more effectively than exposure alone (Kolb et al., 2014; Schön, 1983).
- Preparing to explain an idea—and then actually doing so—solidifies understanding and promotes long-term retention (Fiorella and Mayer, 2013; Nestojko et al., 2014).

In parallel, the principles of collaborative engagement highlighted the value of social interaction: discussing ideas with peers, comparing perspectives, and co-constructing meaning. These interactions deepen comprehension and strengthen the ability to transfer concepts to new contexts (Johnson et al., 1998; Vygotsky, 1980) such as the application of digital twins to biomedicine.

A conference environment was intentionally created to help participants grasp theoretical frameworks and guide them into the practice of using them with real scenarios. By progressing from exposure to interaction to application, the workshop aligned with well-established pathways that help learners retain knowledge, build competence, and translate theory into actionable skill (Anders Ericsson, 2008; Bonwell and Eison, 1991; Chi and Wylie, 2014; Fiore and Schooler, 2004; Fiorella and Mayer, 2013). With a clear structure, the participants would have freedom to explore the BDT space in a manner not dissimilar from the elements provided by an un-conference, T-Groups, the Open Space technology, as well as others (Kleiner, 2008; Lipmanowicz and McCandless, 2013; O’Reilly, 2023; Owen, 2008; Wulf, 2015).

#### 2.1.2 Participant view: seeking collaborative opportunities

As a biomedical scientist studying children’s mental health, I (ZX) had experience collecting and managing diverse data and informing the building of computational infrastructure, yet I recognized that developing a functional biomedical digital twin was beyond the scope of any single discipline. BDTs—digital representations of biomedical systems that model and predict various activities—require diverse disciplinary expertise, advanced computational methods, and real-world clinical applications.

When the National Institutes of Health’s Interagency Modeling and Analysis Group IMAG, a large interagency effort across more than 10 agencies across the U.S. federal government (IMAG, 2025), issued an invitation to the Teaming4BDT conference, I recognized a critical collaborative opportunity. The conference was open to biomedical program directors, academic researchers, and industry partners interested in BDT development, with an explicit focus on applying team science approaches. The agenda was posted online for participants to review ahead of the meeting, so I understood that I was signing up for a conference that would be very interactive and could lead to tangible collaborations. For researchers like me in the early stages of BDT conceptualization, the conference offered three compelling opportunities:

(1) Developing a collective perspective on BDT requirements and evaluation standards.
(2) Understanding the team science principles that create multidisciplinary teams aligned with BDT research goals.
(3) Finding collaborators and forming working teams to develop and pitch BDT ideas under expert guidance while applying team science principles to real-time team dynamics.

Besides allowing participants to learn how BDT teams are formed at the conference, the conference format specifically encouraged diverse multidisciplinary discussions that are important to the application of BDT in children’s mental health, which is not just psychological, but also biological, environmental, and social. BDT in children’s mental health will pave ways for integrating biological, environmental, and social data for safe modeling that would be impossible in real children, personalizing developmental predictions in children, simulating interventions that are lacking in real life, and bridging research and evidence-based care in a continuous feedback loop.

### 2.2 During the meeting

#### 2.2.1 Designer view: implementation

The three-day meeting was structured for moving from ideas to proposing research plans. Early sessions established a shared conceptual grounding in the NASEM definition of a digital twin, using didactic presentations and discussions to build a common vocabulary.

Poster viewing and informal exchanges complemented these sessions by encouraging participants to surface nascent BDT ideas and begin forming connections across disciplinary and organizational boundaries. The alignment of agenda segments with specific learning functions made explicit how the meeting operationalized active, experiential, and collaborative learning principles—from individual understanding through collaborative template development to collective pitch presentations (Table 1).

**Table 1:** Teaming4BDT meeting agenda: learning, design, and intended outcomes.

Agenda item	Learning or design principle	Intended outcome
Day 1		
Welcome; meeting vision & goals	Framing; shared mental model creation	Establish a common purpose and expectations for collaborative learning
Didactic: NASEM digital twin definition	Direct instruction; conceptual scaffolding	Build baseline understanding of digital twin criteria and vocabulary
Panel: unique BDT features	Perspective integration; cognitive contrast	Broaden conceptual frame; surface domain-specific challenges
Breakout: apply definition to own BDT idea	Individual reflection; collaborative meaning-making; structured inquiry	Deepen individual conceptual clarity; practice applying theory to real projects
Poster viewing & idea posting	Informal peer exchange; social learning; idea emergence	Identify shared interests; seed potential team topics
Talks: technical & social components of BDT	Situated didactics; conceptual expansion	Introduce technical and team-science components required for teaming
Breakout #1: generate requirements for BDT	Collaborative construction; use of handouts; iterative refinement	Produce shared BDT requirements; practice operationalizing theory
Open space–style team formation	Emergent collaboration structure; participant-led team formation	Generate 12 team ideas; form self-selected interdisciplinary teams
Day 2		
Requirements template	Whole-group synthesis	Shared understanding
Teaming session #1	Experiential teamwork; template-guided co-creation; flip charts	Begin shaping team BDT concepts; identify assumptions and pitfalls
Poster viewing, idea sharing, networking	Informal peer exchange; social learning; idea emergence	Identify shared interests; nurture team topics, build relationships
Talks: BDT components	Adaptive instruction based on Day 1	Strengthen conceptual foundations
Breakout #2: generate BDT assessment criteria	Collaborative reasoning; criteria development; content generation	Create assessment templates; deepen understanding of maturity and feasibility
Teaming session #2	Iterative design; feedback	Refine models; ID gaps; prep for updates
Team progress reports	Peer teaching; feedback loops; structured critique	Receive targeted feedback; recognize missing expertise; refine direction
Day 3		
Requirements template and assessment criteria	Whole group discussion and synthesis	Establish a shared understanding for deeper team work
Teaming session #3: pitch development	Synthesis; applied collaboration; rehearsal	Translate concept into problem that will be solved with plan and timeline
Pitch sessions	Authentic performance; peer evaluation; applied use of criteria	Test clarity and maturity of ideas; experience evaluator perspective
Full-group debrief	Reflective practice; metacognition; feedback consolidation	Identify enablers and barriers; extract transferable lessons for future teams
Closing & next steps	Transition; forward planning	Pathways for continued collaboration

A visual map of the meeting agenda onto the learning design, illustrating how didactic inputs, collaborative breakouts, structured templates, participant-generated content, and iterative synthesis were sequenced to support conceptual development and team formation. The alignment of agenda segments with specific learning functions makes explicit how the meeting operationalized active, experiential, and collaborative learning principles. The table also identifies some points at which handouts were used, when teams generated materials (e.g., templates, flip charts, recommendations), and when they presented or synthesized their work, showing the progression from individual understanding to collective pitch development. (Meeting agenda accessible at: https://www.imagwiki.nibib.nih.gov/sites/default/files/Teaming4BDT%20Conference%20Booklet.pdf and assessment form at: https://www.imagwiki.nibib.nih.gov/sites/default/files/Pitch%20Assessment%20Form.docx.).

Across Day 2, these self-assembled teams met twice to develop their concepts, integrating insights from requirement-generation and assessment-criteria sessions. Rotating breakout groups, a collaborative tool for note-taking, and shared Google slide templates ensured that teams were continually exposed to diverse perspectives while also producing tangible artifacts of their collective reasoning. By the end of the day, all teams presented brief progress updates that described their projects, key requirements, ethical considerations, and gaps in needed expertise.

On Day 3, 11 teams further developed and delivered structured pitches using a slide template. These sessions required teams to synthesize conceptual, technical, and organizational elements of their proposed BDTs, while audience members provided assessments based on criteria developed earlier in the meeting. The event concluded with a whole-group debrief that surfaced reflections on collaboration, opportunities, challenges, and lessons learned—reinforcing the meeting’s overarching goal of linking learning, practice, and team formation.

#### 2.2.2 Participant view: putting collaborative principles into action

I was aware of four conceptual elements that guided the conference including components, requirements, assessment criteria, and project ideas which at first may seem very complex. Yet, the color-coded agenda for the conference highlighted the progression of sessions, and served as a mental map to better visualize a BDT as research endeavor. The agenda also served as a discussion framework to guide the participants through the joint and breakout sessions.

Enabled by the agenda, all attendees participated actively. Instead of passive listening, the conference allowed time for continuous information exchange between speakers, experts, and the audience (BDT teams). The poster sessions and opportunities to leave messages on the poster boards allowed the participants to ask questions and give feedback to others. Moreover, it helped attendees learn the challenges other BDT researchers encountered and the methods and technologies they applied to overcome these obstacles. From a participant’s perspective, these dialogues stood out as the most memorable and beneficial aspect of this conference, allowing the attendees to know each other, exchange ideas and explore teaming potential. Another unique design of this conference was the self-formation of transient teams and fast pitch of BDT ideas.

At the end of Day 1, focused on the definition and components of BDT, the conference organizers encouraged attendees to share their own BDT research topics with the conference audience. Attendees could either lead or join a BDT idea team based on the alignment of their own interests. As a biomedical scientist, I presented a rough BDT idea on children’s mental health and was encouraged to lead when a number of attendees including computer scientists, biomedical engineers, and biostatisticians expressed that the idea was aligned with their current research to better understand the cause of autism. An interdisciplinary team was formed and our collaboration started.

On Day 2, focused on the approaches to address BDT challenges, attendees learned about the technical and social components of BDT, as well as developed the assessment criteria templates that were used to give and receive feedback on BDT ideas. Based on individual members’ technical expertise on our team, we discussed and agreed on assigning different team members to follow breakout sessions on various topics of BDT. This approach established sub-topic leaders and further defined each member’s responsible areas, allowing each member to focus on a subset of requirements and fully engage in the deep dive on BDT.

On Day 3, we operationalized Team Science for BDT. Our team spent half day putting information we learned together to create a pitch and presented as a team while the audience used the feedback template to evaluate and give our team feedback. In turn, we provided feedback on other teams’ BDT ideas.

This unique transient team exercise mimicked the real-world complex scenario in BDT research. It allowed us to sketch out a prototype of the BDT research plan in a very short time while applying team science principles to address the differences and diverse approaches to reach consensus.

### 2.3 After the meeting

#### 2.3.1 Designer perspective: a meeting like no other

Immediately following the conference, participants offered unsolicited reflections that underscored the impact of the meeting’s design. It was markedly different from traditional scientific conferences, unusually interactive, engaging, and conducive to meaningful learning. Like an intensive short course, the combination of was immediate utility of the tools and frameworks, greater clarity about the expertise required for BDT development, and experience in how to begin planning or advance their own projects.

Transient teams rapidly developed trust and psychological safety—critical conditions for interdisciplinary collaboration (Edmondson, 1999, 2019). The iterative individual reflection, small-group exploration, and visible whole-group synthesis created an environment where participants felt comfortable testing ideas, asking questions, and acknowledging uncertainty. Because teaming was initiated through an open, participant-driven process, many groups relied on “swift trust,” a form of action-oriented trust that allows individuals to collaborate effectively before personal familiarity has developed (Bennett et al., 2025).

The meeting’s facilitation practices normalized diverse expertise, emphasized curiosity over critique, and made contributions visible through templates, flip charts, and iterative feedback. These elements enabled teams to work productively within hours, illustrating the relational foundations necessary for advancing emerging BDT concepts (Bennett et al., 2025).

Early signs of continued collaboration further reinforced the meeting’s impact. Multiple groups expressed intentions to continue refining their ideas, and at least one team—led by Dr. Zhen Xiao—continued meeting after the conference to advance a digital twin concept focused on families navigating autism or ADHD. Participants requested permission to reuse handouts, templates, or teaming structures at their home institutions, indicating transfer of learning and adaptation of the meeting’s design. Collectively, these reflections suggest that the event strengthened conceptual understanding and catalyzed collaborative momentum within the emerging BDT community.

#### 2.3.2 Participant perspective: sustaining collaboration

The intentional design of the Teaming4BDT conference agenda empowered our team’s sustained collaborations post conference. The following summarizes the follow-up development of the co-author Dr. Xiao’s team and the collaborative principles that were used.

- *Agree on the team vision*: our interdisciplinary team met again after the conference to further build trust and reiterate the joint interest to continue our collaboration on the BDT idea. We reflected on the BDT research challenges and opportunities from the conference. We realized a cohesive vision and strategy over the long term will help each team member as well as our team to achieve research goals.
- *Expand the team*: we expanded our interdisciplinary collaboration to include patient advocates, technology developers, and service providers who brought in additional expertise and insights in BDT. We also include trainees and mentees into our collaborative efforts to cultivate their skills and experience in team science as well as BDT research.
- *Define roles*: we built up mutual understanding of roles and responsibilities. Each team member became a leader in a respective sub-topic on our team, including scientific, technical, social and ethical aspects of BDT. This team line-up and organizational development occurred naturally after conference because of the common understanding of the team science principles we learned and our shared desire to practice them intentionally.
- *Respond to research opportunities*: 6 months after the conference, our team was presented with an opportunity to respond to a research initiative with a very compressed timeline. Within weeks, we assembled and submitted a research proposal that included a full technical proposal, letters of support, community engagement plan, data management plan, key personnel bio sketches, as well as research budget. While the team did not receive funding for the full proposal, Dr. Xiao was accepted to and funded for a leadership fellowship in the related field. This allowed her to continue to develop the team and carry out the proposed community engagement plan to better prepare for future research opportunities.
- *Maintain communications and external outreach*: The collective strength of a team is built upon the communications among members. We maintain internal communications to keep each other updated on research progress and career movements. We also continue to attend other BDT related conferences as part of our outreach efforts.

More than a year after the Teaming4BDT conference, our team continues to benefit from the knowledge, expert guidance, and the solid collaboration framework we gained from the 3-day event. We still consider our team in the stage of forming as the collaboration circle must grow to meet the challenges of complex BDT. We had a taste of storming when responding to a major research opportunity. Our team’s journey towards norming, performing and transforming will continue (Tuckman, 1965; Tuckman and Jensen, 1977).

## 3 Discussion

Through dual perspectives—designer and participant—we illustrated how a conference can be intentionally structured for participants to practice teaming around challenges that require an interdisciplinary approach. The design provided a framework that moved participants from theoretical understanding of BDTs and the collaboration necessary for application. We documented one participant’s journey from team formation at the conference through proposal development and ongoing partnership. These perspectives reveal how deliberate conference design lowers barriers to collaboration and transforms abstract concepts into actionable research partnerships.

Iterating didactic content with collaborative inquiry, structured templates, and authentic practice succeeded in helping participants navigate the intellectual and organizational complexity inherent in BDT development. The participant experience confirmed these intentions: the color-coded agenda provided a mental map for navigating BDT complexity, the poster and breakout sessions facilitated meaningful dialogue, and the transient team exercise mimicked real-world collaborative scenarios in compressed time.

The shared frameworks and team science principles became the foundation for Dr. Xiao’s team’s continued work—guiding role definition, expanding membership, respondes to funding opportunities, and effective communication. The rapid assembly of a comprehensive research proposal, securing a leadership fellowship, and sustained engagement suggests that the conference’s scaffolding equipped participants with transferable tools for long-term collaboration. The team’s progression through Tuckman’s stages (forming, storming, norming and performing) was guided by the shared language and collaborative practices promoted at the three-day event (Tuckman, 1965).

Broadly, this dual perspective reveals three transferable elements for conferences aiming to accelerate field-building in emerging areas:

(1) Scaffolding conceptual learning with authentic practice. Design: structured progression from didactics to application. Participant: sketched a BDT research plan while applying newly learned principles in real time.
(2) Catalyzing “productive collisions” across disciplines: Design: intentionally used Open Space methods and rotating breakouts. Participant: memorable dialogues that allowed networking idea exchange, and organic teaming potential.
(3) Translating abstract frameworks into concrete work products. Design: slide templates, assessment criteria, and pitch structures made thinking visible. Participant: these same tools became artifacts they could adapt and reuse in their work.

Deliberately structuring a conference as a learning and teaming event can serve as powerful foundation for catalyzing collaboration. The participant perspective confirms what the design aimed to achieve: not just knowledge transfer, but the development of an opportunity extending beyond the event itself.

Given the need for disciplinary integration to solve complex societal problems, this model offers a repeatable approach for supporting researchers as they learn, team, and co-develop shared standards. Future events may build on these lessons to further strengthen infrastructure for training, teaming, and innovation—not only in BDT research, but across emerging scientific domains where interdisciplinary collaboration remains more aspirational than operational.

Several limitations merit consideration. We document only one team’s sustained journey in detail, and the team was self-selected, potentially representing participants already predisposed to interdisciplinary work. The conference design also required significant collaborative facilitation expertise (Bennett and Wilkinsky, 2025), structured preparation time, and willingness to reduce formal presentation slots in favor of collaborative workspace—resources not available to all conference organizers.

Many emerging scientific fields require active team formation and conferences can be designed as more than just presentation venues. While the focus of this meeting was on BDTs, the design can be applied to any interdisciplinary research field. A modest investment in structured design can move people and teams from knowledge gain to practice.

## Data Availability

The original contributions presented in the study are included in the article, further inquiries can be directed to the Michelle.LMBC@gmail.com.
